# A survey and analysis of peri-operative quality indicators promoted by National Societies of Anaesthesiologists in Europe: The EQUIP project

**DOI:** 10.1097/EJA.0000000000002054

**Published:** 2024-09-12

**Authors:** Johannes Wacker, Guy Haller, Jan F.A. Hendrickx, Martin Ponschab

**Affiliations:** From the University of Zurich, Faculty of Medicine, Zurich, Switzerland (JW), Institute of Anesthesia and Intensive Care, Hirslanden Clinic, Zurich (JW), Department of Acute Care Medicine, Division of Anesthesiology, Geneva University Hospitals and Faculty of Medicine, University of Geneva, Geneva, Switzerland (GH), Department of Epidemiology and Preventive Medicine, Health Services Management and Research Unit, Monash University, Melbourne, Victoria, Australia (GH), Department of Anesthesiology, Intensive Care and Pain Therapy, OLV Hospital, Aalst (JFAH), Department of Basic and Applied Medical Sciences, Ghent University, Ghent (JFAH), Department of Anesthesiology, UZLeuven, Leuven, Belgium & Department of Cardiovascular Sciences, KULeuven, Leuven, Belgium (JFAH), Ludwig Boltzmann Institute for Experimental and Clinical Traumatology, AUVA Research Centre, Vienna (MP), Department of Anesthesiology and Intensive Care, AUVA Trauma Hospital Linz, Academic Teaching Hospital of the Paracelsus Medical University, Salzburg, Austria (MP)

## Abstract

**BACKGROUND:**

To capture preventable peri-operative patient harm and guide improvement initiatives, many quality indicators (QIs) have been developed. Several National Anaesthesiologists Societies (NAS) in Europe have implemented quality indicators. To date, the definitions, validity and dissemination of such quality indicators, and their comparability with validated published indicators are unknown.

**OBJECTIVES:**

The aim of this study was to identify all quality indicators promoted by NAS in Europe, to assess their characteristics and to compare them with published validated quality indicators.

**DESIGN:**

A cross-sectional study with mixed methods analysis. Using a survey questionnaire, representatives of 37 NAS were asked if their society provided quality indicators to their members and, if so, to provide the list, definitions and details of quality indicators. Characteristics of reported quality indicators were analysed.

**SETTING:**

The 37 NAS affiliated with the European Society of Anaesthesiology and Intensive Care (ESAIC) at the time. Data collection, translations: March 2018 to February 2020.

**PARTICIPANTS:**

Representatives of all 37 NAS completed the survey.

**MAIN OUTCOME MEASURES:**

QIs reported by NAS.

**RESULTS:**

Only 12 (32%) of the 37 NAS had made a set of quality indicators available to their members. Data collection was mandatory in six (16.2%) of the 37 countries. We identified 163 individual quality indicators, which were most commonly descriptive (60.1%), anaesthesia-specific (50.3%) and related to intra-operative care (21.5%). They often measured structures (41.7%) and aspects of safety (35.6%), appropriateness (20.9%) and prevention (16.6%). Patient-centred care (3.7%) was not well covered. Only 11.7% of QIs corresponded to published validated or well established quality indicator sets.

**CONCLUSIONS:**

Few NAS in Europe promoted peri-operative quality indicators. Most of them differed from published sets of validated indicators and were often related to the structural dimension of quality. There is a need to establish a European-wide comprehensive core set of usable and validated quality indicators to monitor the quality of peri-operative care.

**TRIAL REGISTRATION:**

No registration.

**KEY POINTS**

- Peri-operative quality improvement is urgent, and quality indicators are efficient tools to address quality and safety issues in anaesthesia.
- To date, there is no unified and validated set of peri-operative quality indicators in use in Europe.
- This study found that only a few National Anaesthesiologists Societies in Europe promoted peri-operative routine quality indicators, which were rarely comparable with validated quality indicator sets, and omitted relevant quality aspects.
- The results can be used for future research and practice development aiming to establish a core set of valid and practicable peri-operative quality indicators.

## Introduction

Despite numerous patient safety and quality initiatives over the past decades,^1–6^ preventable patient harm remains a serious risk in peri-operative care.^7,8^ Peri-operative complications are reported to occur in 19 to 40% of surgical cases,^7,9,10^ and peri-operative mortality rates range from 1.2 to 21.5%.^9–13^ Therefore, preventing harm and improving the overall quality of care remains a priority. Several tools have been developed in order to monitor quality and to implement quality improvement.^14,15^ The best known are quality indicators.^16,17^ They offer the opportunity to improve patient safety and outcomes according to defined criteria in specific areas.^18,19^ However, quality indicators have several limitations.^20^ One is the multiplicity of measures needed to describe the different aspects of peri-operative care, and to capture its full complexity.^16^ Another is the unclear validity of these measures in clinical practice.^16^ Finally, using quality indicators as improvement tools may require time,^21^ additional funding^20^ and appropriate staffing.^21^

Despite these shortcomings, several National Anaesthesiologists Societies (NAS) in Europe and other organisations have developed quality indicators and promoted their use to improve peri-operative care.^22–26^ The number, validity and usability of these NAS-recommended quality indicators are currently unknown. Recognising this problem, the Patient Safety and Quality Committee (PSQC) of the European Society of Anaesthesiology and Intensive Care (ESAIC) initiated the ESAIC Quality Indicator Project (EQUIP).^27^

The aim of EQUIP was to identify all quality indicators promoted by NAS in Europe, to assess their characteristics, usability and validity, and to compare them with quality indicators developed in other countries, by other research groups, and published in the English medical literature.^16,27–29^ This approach may help define a comprehensive core set of operational and valid quality indicators, allowing more accurate appraisal and monitoring of peri-operative quality and safety.^27^

## Materials and methods

### Ethics approval

Ethics approval was not required,^30^ as no original human or animal data were used.^31^

### Study design

We conducted a cross-sectional survey study. A standardised questionnaire was sent to all NAS representatives asking for the names and definitions of the indicators recommended by their society. No restrictions applied. The collected indicators were then rated, classified and analysed using mixed methods.^32^ The SQUIRE guideline items were considered and adapted to the study design.^33,34^

### Study organisation, questionnaire and selection of respondents

**Fig. 1:**
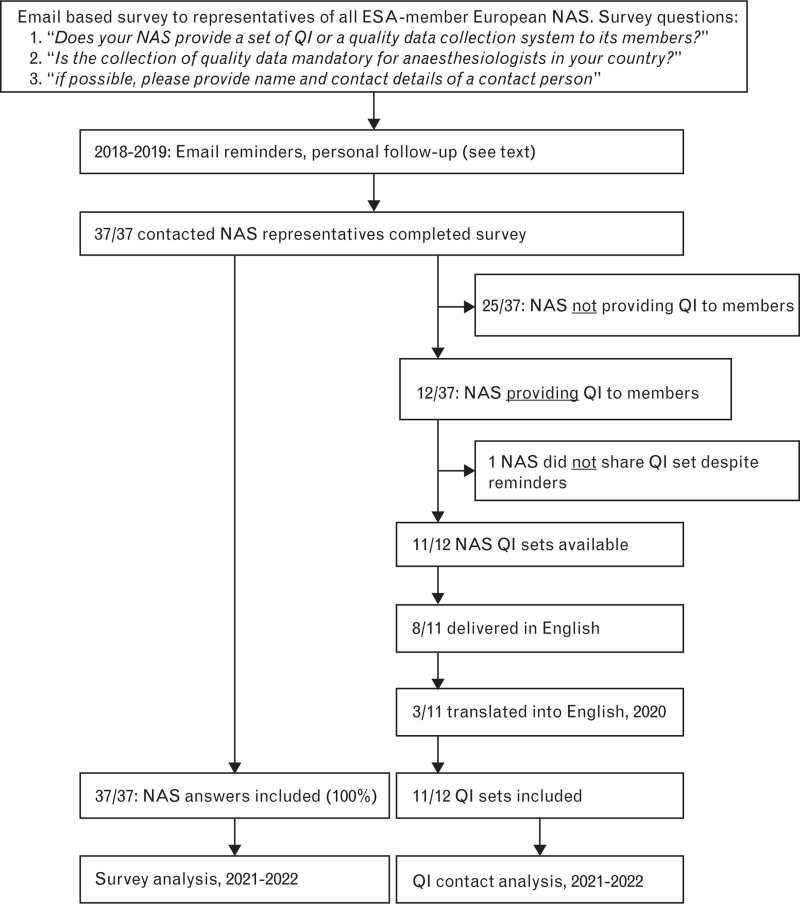
EQUIP survey of NAS representatives – flow chart.

The EQUIP team, consisting of members of the ESAIC PSQC, prepared an initial project draft that was approved by the ESAIC Board of Directors and the NAS Committee Chair. All NAS representatives were informed about the survey by E-Mail and during the 2016 Euroanaesthesia conference. The final survey was then formalised (survey questions: Fig. 1). NAS representatives were identified using a list of ESAIC member NAS at that time provided by the ESAIC secretariat. E-Mail, telephone and face-to-face contacts during professional meetings (e.g. Euroanaesthesia) were used to attain a high response rate. NAS and country names were anonymised in the final results. The follow-up process and the COVID-19 pandemic extended the duration of this study.

EQUIP, ESAIC Quality Indicator Project; ESA (now ESAIC), European Society of Anaesthesiology and Intensive Care; NAS, National Anaesthesiologists Society; NASC, National Anaesthesiologists Societies Committee (NASC); QI, quality indicator; contact person, person to be contacted regarding QIs and quality topics; left column, analysis of survey questions; right column, analysis of QIs.

### Document and data management

Quality indicator sets submitted in other languages were translated into English. For this purpose, they were contracted by the ESAIC secretariat to a translation agency (Viaverbia Bruxelles, https://www.viaverbia.lu). Two authors (JW, MP) reviewed and discussed both translated and original language quality indicator sets. Translations were adapted when the meaning could be improved considering the anaesthesia context (list of adaptations: Supplemental Digital Content (SDC) 1). Primary survey responses and quality indicator information were screened, extracted and transcribed into Microsoft Excel spreadsheets by one researcher (JW) and checked for errors and inconsistencies by a second researcher (MP).

### Analysis criteria and rating variables

For the analysis and classification of quality indicator characteristics, we used 21 criteria that have been used in previous studies on this topic^1,16,20,28,29,35,36^ (list of detailed criteria: SDC 2): a unique quality indicator identifier; name or title of the quality indicator provided by the NAS; detailed description or information (if provided); type of indicator (descriptive, prescriptive, proscriptive);^16^ area of care (process, outcome or structure according to Donabedian's model of quality of care ^37^); peri-operative phase (pre-operative, intra-operative, postoperative^29^ or ’not applicable’); anaesthesia specific or general; and^16^ dimensions of quality (10 different attributes according to the National Library of Healthcare Indicators,^16^ or ’other dimension’). We also assessed the validity of the quality indicators by comparing them with quality indicator sets validated for research purposes^20,28,35^ or clinical use ^29,36^ or representing consensus quality indicators used in specific clinical settings: The StEP Consensus Clinical Indicators (for use in clinical trials^28^); Peri-operative Patient Safety Indicators (clinically tested in hospitals ^36^); Structure quality indicators^29^ with scientific level 1 evidence;^38^ Process Indicators^29,36^ with scientific level 1 evidence;^38^ StEP Patient Centered Outcomes (for clinical trials)^35^; 18 ’Principal Requirements’ of the Declaration of Helsinki on Patient Safety in Anaesthesiology^1^ (similar to well accepted Structure quality indicators).

Country-specific variables were also included: countries of reporting NAS were classified according to the physician anaesthesia provider (PAP) density published by the World Federation of Societies of Anaesthesiologists (WFSA),^39,40^ the World Bank Gross National Income (WBGNI) groups^41^ and the geographical regions defined by the United Nations Statistical Division.^42^

### Rating process and consensus rating discussions

Two authors (JW, MP) independently assessed all quality indicators reported by NAS against the 21 criteria variables defined above. They assessed whether the quality indicators measured the same concept as the compared criteria. The raters had many years of clinical experience as consultant anaesthetists and extensive theoretical and practical expertise in peri-operative safety and quality management. Initial inter-rater agreement was measured, and disagreements were discussed^16^ until consensus was reached.

### Additional analyses

We supplemented the formal expert-based validation process with mixed methods to optimise the breadth and depth of the analysis:^32^ as an embedded qualitative approach,^32^ word frequency counting and word cloud graphing were used, as well as thematic evaluation to identify and visualise prominent themes.^43^ We looked more specifically at quality indicators that did not match previously published indicator sets.^1,28,29,35,36^ MAXQDA ^44^ was used to generate lists of single words and two-word combinations (minimum frequency: 2) using a standard ‘stop word list’. We excluded commonly used words and filtered out hyperlinks, E-Mail addresses, hashtags, numbers and text within square and curly brackets.^44,45^ A number of generic terms (e.g. ‘anaesthesia’, ‘anaesthesia’, ‘system’) were also excluded from the generation of word clouds to focus on the practical aspects of care described by the quality indicator. In addition, informal observations of quality indicator characteristics made during the ratings and consensus meetings were included in the assessment.

### Statistical analysis

For analyses of survey data, the results for categorical variables were summarised as frequencies and percentages.^45^ We used risk ratio calculations and chi-squared tests for independence^46^ to assess the relationship between the independent variables (mandatory reporting, WFSA PAP density, WBGNI group and Western/Northern Europe versus other regions) and the binary outcome of whether or not NAS provided a quality indicator set to their members and to assess differences between quality indicator subsets. Interrater reliability was evaluated by calculating both percentage agreement and kappa statistics^47–49^ with confidence intervals.^50^ In the rare case of 100% interrater agreement in Excel, the kappa statistic was not calculated.^49^ Strengths of agreement were defined as follows:^51^ poor: < 0.00; slight: 0.00 to 0.20; fair: 0.21 to 0.40; moderate: 0.41 to 0.60; substantial: 0.61 to 0.80; and almost perfect: 0.81 to 1.00.^51^ Word frequencies and word clouds were generated using MAXQDA^44^ (MAXQDA Analytics Pro 2022, VERBI Software GmbH, Berlin, Germany). Other software included Preview, Microsoft Excel and Word for Mac, EndNoteTM X8.2 and Stata/IC 16.1 for Mac (Intel 64-bit), Revision 14 June 2022, Copyright 1985–2019 StataCorp LLC, College Station, Texas, USA.

## Results

### Survey of National Anaesthesiologists Society representatives

The response rate was 100% (Fig. 1 and Table 1). We found that quality data collection was mandatory for anaesthesiologists in only six (16.2%) of the 37 European countries surveyed. Twelve (32%) of the NAS made a quality indicator set available to their members, and 11 of them shared their quality indicator set with the EQUIP study team. Eight sets were provided in English, and the remaining three sets were translated into English. Within the translated sets, 13 individual QIs (8%) required clarification (SDC 1). Transcriptions of quality indicator information were reviewed and released for classification after meaningful adaptation to the anaesthesiology context and double-checking.

**Table 1:** Survey overview and countries of participating National Anaesthesiologists Societies

Variables	No. of NAS	Providing QI sets to members *n* (%)	Not providing QI sets to members *n* (%)
UN geographic region^42^ of countries with participating NAS	37	12 (32.4)	25 (67.6)
Western Europe	6	5 (83.3)	1 (16.7)
Northern Europe	8	3 (37.5)	5 (62.5)
Eastern Europe	9	1 (11.1)	8 (88.9)
Southern Europe	10	2 (20)	8 (80)
Western Asia	4	1 (25)	3 (75)
WFSA Physician anaesthesiologist workforce density of NAS country^39,40^	36	12 (33.3)	24 (66.7)
Physician anaesthesia provider (PAP) density < 10/100’000	6	2 (40)	4 (60)
PAP density 10–< 20/100’000	19	4 (21.1)	15 (78.9)
PAP density 20–< 30/100 000	8	2 (25)	6 (75)
PAP density > 30/100 000	4	4 (100)	0 (0)
WBGNI Group of NAS country (June 2020)^41^	37	12 (32.4)	25 (67.6)
Low income (≤ $1035)	0	0 (NA)	0 (NA)
Lower middle income ($1036 to 4045)	2	0 (NA)	2 (100)
Upper middle income ($4046 to 12 535)	7	0 (NA)	7 (100)
High income (≥ $12 536)	28	12 (42.9)	16 (57.1)

NAS of the following 37 countries answered the survey (response rate 37/37 = 100%): Austria; Belgium; Bulgaria; Croatia; Cyprus; Czech Republic; Denmark; Estonia; Finland; France; Georgia; Germany; Greece; Hungary; Israel; Italy; Kosovo; Latvia; Lithuania; Malta; Moldova, Republic Of; Netherlands; Norway; Poland; Portugal; Republic of Macedonia; Romania; Russian Federation; Serbia; Slovakia; Slovenia; Spain; Sweden; Switzerland; Turkey; Ukraine; UK and Ireland. The WFSA PAP density value is missing in the reference for one country of our analysis, hence the total of 36. NAS, National Anaesthesiologists’ Society; *N*, number; PAP, physician anaesthesia provider; QI, quality indicator; UN, United Nations; WBGNI, World Bank Gross National Income; WFSA, World Federation of Societies of Anaesthesiologists.

### Predictors of National Anaesthesiologists Society providing a quality indicator set to members

We found the following positive predictors: mandatory reporting of quality data in the respective country [risk ratio = 5.167 (95% CI 2.519 to 10.599); χ^2^ = 14.92, *P* = 0.000]; country located in Western or Northern Europe (versus other regions)^42^ [risk ratio = 3.286 (95% CI 1.209 to 8.928); χ^2^ = 6.28, *P* = 0.012]; density of physician anaesthesia providers^39,40^ in the respective country greater than or equal to 25 per 100 000 inhabitants (risk ratio = 3.690 (95% CI 1.754 to 7.763); χ^2^ = 8.47, *P* = 0.004]; and all NAS providing quality indicator sets were from WBGNI high-income countries.^41^

### Heterogeneity of quality indicator sets

The 11 quality indicator sets shared by NAS and included in the study were very heterogeneous, containing a total of 163 individual quality indicators (list of quality indicator names or titles: SDC 3). The size of individual sets ranged from 2 to 41 quality indicators, with a median of 13 [IQR 9 to 17]. Quality indicators were defined very unevenly: in some cases, a quality indicator represented a complex set of criteria; in others, it represented only one basic variable. One NAS submitted a member survey that included quality indicators with more than 90% agreement among respondents but that had not yet been implemented. These were included in the study by consensus of the assessors.

### Rater-based classification of National Anaesthesiologists Society reported quality indicators

The 163 quality indicators were rated according to 21 criteria (see above). Thus, the raters initially completed 3423 individual ratings independently. They then discussed 686 disagreements (20.04%). Consensus ratings were used for the analysis.

### Characteristics of National Anaesthesiologists Society reported quality indicators

We found that after rater consensus, the most common type of quality indicator was descriptive (60.1% of quality indicators; Table 2). Quality indicators were more evenly distributed across care settings, with structural quality indicators being the most common (41.7%). Quality indicators were most frequently related to the intra-operative phase (21.5%), but 45.4% could not be related to a specific phase of peri-operative care. Anaesthesia-specific (50.3%) and general quality indicators (49.7%) were equally distributed.

**Table 2:** Descriptive characteristics of quality indicators reported by National Anaesthesiologists Societies

Measuring aspect (definition)^16^	*n* (% QI)	Example of QI reported by NAS
Type of indicator^16^		
Descriptive (descriptive information on unusual situations of patient care that could reveal, if further investigated, potential defects in the quality of care provided - e.g. unplanned overnight admission of day surgery patients for anaesthetic reasons)	98 (60.1)	*‘Unplanned postoperative transfer (to the intensive care unit; directly from OT)’*
Prescriptive (indicator represents recommendations or desired targets - e.g. prophylactic antibiotic selection for surgical patients according to current recommendations)	46 (28.2)	*‘Timely administration (30–60 min before incision) of antibiotics in those cases needing antibiotics’*
Proscriptive (measures of actions that ‘should not be performed’ - e.g. medication error with the wrong medication being given)	19 (11.7)	*‘Number of requests for labour analgesia not satisfied within 30 and 60 Minutes’*
Area of care^37^ described by the QI		
Process (indicator refers to implementation of programme activities)	44 (27)	*‘Pain measurement on the ward, at least 3 times a day NRS taken and documented’*
Outcome (indicator refers to patient-related end results of anaesthesia (peri-operative) care)	51 (31.3)	*‘Death < 48h after anaesthesia’*
Structure (indicator refers to hospital staff, material and overall organisation)	68 (41.7)	*‘Number of surgical beds’*
Peri-operative phase^29^ described by the QI		
Pre-operative (from the decision to operate to entry into the theatre suite)	25 (15.3)	*‘Preoperative patient evaluation for anaesthesia: ASA Classification’*
Intra-operative (from entry into the theatre suite to leaving the recovery area)	35 (21.5)	*‘Anaesthesia related problems - Death during anaesthesia’*
Postoperative (following transfer from the recovery area)	29 (17.8)	*‘Postoperative visit of the patient’*
Not applicable	74 (45.4)	*‘Number of complaints per 1000 anaesthetic procedures’*
QI describing anaesthesia-specific or general^16^ aspects of care		
Specific (indicator refers specifically to the practice of anaesthesia)	82 (50.3)	*‘Intraoperative - Anaesthesia equipment checklist’*
General (indicator could also relate to surgical or postoperative ward care)	81 (49.7)	*‘Reporting about perioperative mortality and morbidity on a yearly basis as well as measures for improvement’*

QI, quality indicator; % QI, % of all reported 163 QI; ASA, American Society of Anesthesiologists; *n*, number of QI; NAS, National Anaesthesiologists Society; NRS, numeric rating scale; OT, operating theatre.

### Attributes of quality reflected by National Anaesthesiologists Society reported quality indicators

Quality indicators were classified according to 10 quality subdimensions^16^ (Table 3). We found that quality indicators were most often related to the safety dimension of care (35.6%), followed by appropriateness (20.9%) and prevention (16.6%).

**Table 3:** Dimensions of quality reflected by quality indicators reported by National Anaesthesiologists’ Societies

Subdimension^16^ (definitions)	*n* (% QI)	Example of QI reported by NAS
1. Appropriateness (the degree to which the care provided is relevant to the patient's clinical needs, given the current state of knowledge)	34 (20.9)	*‘The proportion of patients who were discharged from a recovery room when they reported a VAS score of 3 or less’*
2. Availability/accessibility (the degree to which appropriate care is available to meet the patient's needs)	6 (3.7)	*‘Number of requests for labour analgesia not satisfied within 30 and 60 minutes’*
3. Continuity (the degree to which the care for the patient is coordinated among practitioners, organisations and over time)	3 (1.8)	*‘Postoperative care (up to max. 72h): RA-catheter support (1x/24h)’*
4. Effectiveness (the degree to which care is provided in a correct manner, given the current state of knowledge, to achieve the desired or projected outcome(s) for the patient)	21 (12.9)	*‘Preoperative patient evaluation for anaesthesia: Determining the metabolic equivalents (METS)’*
5. Efficacy [the degree to which the care of the patient has been shown to accomplish the desired or projected outcome(s)]	22 (13.5)	*‘Postoperative: Fraction of patients with nausea requiring treatment in the postoperative observation unit, indicator standard < 2%’*
6. Efficiency (the relationship between the outcomes (results of care) and the resources used to deliver patient care)	10 (6.1)	*‘Operating sessions ended after the scheduled time (> 60 min)’*
7. Prevention (the degree to which appropriate services are provided for promotion, preservation, and restoration of health and for early detection of disease)	27 (16.6)	*‘Existence and use of a list for the daily control of ventilation equipment (respirator)’*
8. Respect and caring (the degree to which a patient, or designee, is involved in his or her own care decisions, and to which those providing services do so with sensitivity and respect for the patient's needs, expectations and individual differences. This dimension represents patients’ perspectives)	6 (3.7)	*‘Postoperative - patient satisfaction’*
9. Safety (the degree to which adverse outcomes or injuries stemming from the processes of healthcare are reduced or avoided for the patient)	58 (35.6)	*‘Reporting about perioperative mortality and morbidity on a yearly basis as well as measures for improvement’*
10. Timeliness (the degree to which the care is provided to the patient at the most beneficial or necessary time)	6 (3.7)	*‘Perioperative procedural time’*
Other dimension	0 (0)	

Subdimensions of quality represented by NAS-reported QIs (10 different quality attributes according to National Library of Healthcare Indicators,^16^ or ‘other dimension’). METS, metabolic equivalent (of task); NAS, National Anaesthesiologists Society; *n*, number of QI; % QI, % of all reported 163 QI; QI, quality indicator; RA, regional anaesthesia; VAS, visual analogue scale.

### Comparison of National Anaesthesiologists Society reported quality indicators with validated or well established quality indicator sets

Nineteen quality indicators (11.7%) matched at least one existing published quality indicator set (Tables 4 and 5), with four of these matching two published sets. Common topics among NAS quality indicators that matched published quality indicator sets were pre-operative antibiotic administration (4x); surgical safety checklist (3x); and morbidity and mortality registry/reporting (2x).

**Table 4:** Comparisons of National Anaesthesiologists Society reported quality indicator with validated or well established quality indicator sets

Compared validated QI set	*N* (% of QI)	Example NAS QI	Matching validated QI/item
StEP consensus clinical indicators (7 items)^28^	1 (0.6)	*‘Operative anaesthesia: Unplanned transfer to the ICU’*	Admission to the ICU within 14 days of surgery
PSI patient safety indicators (11 indicators)^36^	6 (3.7)	*‘Intraoperative - antibiotic prophylaxis’*	Timely administration of antibiotic prophylaxis (% patients)
Structure QI with high level of evidence (seven indicators)^28,29,38^	1 (0.6)	*‘Number of beds’*	Bed size of hospital: How many adult inpatient/overnight/23 h stay available within the hospital
Process QI with high level of evidence (35 indicators)^29,36,38^	4 (2.5)	*‘Preoperative - anaesthesia evaluation in the day before the procedure’*	Percentage of patients who have received an anaesthetic assessment before the day of surgery
StEP patient-centred outcomes (three outcomes)^35^	0 (0)	*N/A*	N/A
Principal requirements, ‘Helsinki Declaration’ (18 requirements)^1^	11 (6.7)	*‘WHO-safe-surgery checklist’*	Support WHO SSSL initiative and checklist

Multiple matches were possible: 23 matches were observed between 19 NAS-reported QIs and validated/established QI sets. Helsinki Declaration, Helsinki Declaration on Patient Safety in Anaesthesiology^1^; *n*, number of matching QI; N/A, not applicable; NAS, National Anaesthesiologists Society; QI, quality indicator; % QI, % of all reported 163 QI; PSI, patient safety indicators^36^; StEP, standardised endpoints in peri-operative medicine^28^; WHO SSSL, WHO ‘Safe Surgery Saves Lives’ initiative.^1^

**Table 5:** Individual quality indicators reported by National Anaesthesiologists’ Societies that matched validated or established quality indicator sets

QI reported by NAS	Compared QI sets	Matching item of compared QI set
*‘Operative anaesthesia: Unplanned transfer to the ICU’*	StEP^28^	Admission to the ICU within 14 days of surgery
*‘Operative anaesthesia: Preoperative timely administration (30 min preop) of antibiotics(?)’*	PSI^36^	Timely administration of antibiotic prophylaxis (% patients)
*‘Safety protocols as proposed in the Helsinki Declaration’*	HD^1^	Covering multiple HD requirements (in the raters view)
*‘Temperature management’*	PROC^29,36,38^	Adults having surgery under general or regional anaesthesia have normothermia (temperature > 36.0°C) maintained before, during and after surgery
*‘WHO-safe-surgery checklist’*	HD^1^	Support WHO SSSL initiative and checklist
*‘Reporting about perioperative mortality and morbidity on a yearly basis as well as measures for improvement’*	PSI^36^	Presence of a morbidity and mortality registration
	HD ^1^	Collect the required data to be able to produce an annual report on patient morbidity and mortality
*‘Antibiotics given within the hour (preferably 30 min) before skin incision’*	PSI^36^ PROC^29,36,38^	Timely administration of antibiotic prophylaxis (% patients) Prophylactic antibiotics are administered within 60 min before start of surgery
*‘Presence of anaesthetic examination documentation for elective surgery’*	HD^1^	Should have protocols, and the necessary facilities for managing the following: Pre-operative assessment and preparation
*‘No of beds’*	STRUCT^29,38^	Bed size of hospital: How many adult inpatient/overnight/23 h stay available within the hospital
*‘Do you have surgical check-list?’*	HD^1^	Support WHO SSSL initiative and checklist
*‘Are you labelling syringes?’*	HD^1^	Should have protocols, and the necessary facilities for managing the following: Syringe labelling
*‘Do you have critical accident reporting?’*	HD^1^	Contribute to recognised audits of safe practice and critical incident reporting systems. Resources must be provided to achieve this
*‘Postoperative analgesia: Methods; drugs; do you have multimodal analgesia protocols? Do you use NRS, VAS?’*	HD^1^	Should have protocols, and the necessary facilities for managing the following: Postoperative care including pain relief
*‘Timely administration (30–60 min before incision) of antibiotics in those cases needing antibiotics’*	PSI^36^ PROC^29,36,38^	Timely administration of antibiotic prophylaxis (% patients) Prophylactic antibiotics are administered within 60 min before start of surgery
*‘Preoperative - anaesthesia evaluation in the day before the procedure’*	PROC^29,36,38^	Percentage of patients who have received an anaesthetic assessment before the day of surgery
*‘Intraoperative - surgical safety checklist’*	HD^1^	Support WHO SSSL initiative and checklist
*‘Intraoperative - difficult airways units’*	HD^1^	Should have protocols, and the necessary facilities for managing the following: Difficult/failed intubation
*‘Intraoperative - antibiotic prophylaxis’*	PSI^36^	Timely administration of antibiotic prophylaxis (% patients)
*‘Postoperative - morbidity and mortality registry’*	PSI^36^	Presence of a morbidity and mortality registration
	HD^1^	Collect the required data to be able to produce an annual report on patient morbidity and mortality

Multiple matches were possible: 23 matches were observed between 19 NAS-reported QIs and validated/established QI sets. HD, Helsinki Declaration on Patient Safety in Anaesthesiology^1^; NAS, National Anaesthesiologists Society; NRS, numeric rating scale; PROC, process QI with high level of evidence^29,36,38^; PSI, PSI patient safety indicators^36^; QI, quality indicator; StEP, standardised endpoints in peri-operative medicine^28^; STRUCT, structure QI with high level of evidence^29,38^; VAS, visual analogue scale; WHO SSSL, WHO 'Safe Surgery Saves Lives’ initiative^1^. Wording reflects the original word choices in the cited reported QIs or compared QI sets, respectively.

Subgroup analysis highlighted several properties of ‘matching’ versus ‘nonmatching’ quality indicators (Table 6). ‘Matching’ quality indicators were significantly more often *prescriptive* (instead of descriptive or proscriptive), describing *structure* (instead of outcome or process), related to *general* aspects (instead of anaesthesia-specific aspects) and to *safety* (instead of not safety-related) but significantly less often related to *prevention*.

**Table 6:** Differences between QI subsets reported by NAS matching/nonmatching validated QI sets

Variables	Not-matching QIs n (%)	Matching QIs n (%)	Pearson χ^2^ value	*p* value
Indicator type	144 (100)	19 (100)	χ^2^(2) = 33.4281	0.000^∗^
Descriptive	95 (66)	3 (15.8)		
Prescriptive	30 (20.8)	16 (84.2)		
Proscriptive	19 (13.2)	0 (0)		
Area	144 (100)	19 (100)	χ^2^(2) = 6.3275	0.042^∗^
Outcome	48 (33.3)	3 (15.8)		
Process	41 (28.5)	3 (15.8)		
Structure	55 (38.2)	13 (68.4)		
Phase	144 (100)	19 (100)	χ^2^(3) = 3.6076	0.307
Pre-operative	20 (13.9)	5 (26.3)		
Intra-operative	31 (21.5)	4 (21.1)		
Postoperative	28 (19.4)	1 (5.3)		
Not applicable	65 (45.1)	9 (47.4)		
Anaesthesia specific	144 (100)	19 (100)	χ^2^(1) = 4.9517	0.026^∗^
General	67 (46.5)	14 (73.7)		
Specific	77 (53.5)	5 (26.3)		
Dimension measured				
Appropriateness	27 (18.8)	7 (36.8)	χ^2^(1) = 3.3282	0.068
Not	117 (81.3)	12 (63.2)		
Availability	5 (3.5)	1 (5.3)	χ^2^(1) = 0.1518	0.697
Not	139 (96.5)	18 (94.7)		
Continuity	3 (2.1)	0 (0)	χ^2^(1) = 0.4033	0.525
Not	141 (97.9)	19 (100)		
Effectiveness	16 (11.1)	5 (26.3)	χ^2^(1) = 3.4574	0.063
Not	128 (88.9)	14 (73.7)		
Efficacy	21 (14.6)	1 (5.3)	χ^2^(1) = 1.2488	0.264
Not	123 (85.4)	18 (94.7)		
Efficiency	9 (6.3)	1 (5.3)	χ^2^(1) = 0.0284	0.866
Not	135 (93.8)	18 (94.7)		
Prevention	15 (10.4)	12 (63.2)	χ^2^(1) = 33.7833	0.000^∗^
Not	129 (89.6)	7 (36.8)		
Respect and caring	6 (4.2)	0 (0)	χ^2^(1) = 0.8219	0.365
Not	138 (95.8)	19 (100)		
Safety	44 (30.6)	14 (73.7)	χ^2^(1) = 13.6213	0.000^∗^
Not	100 (69.4)	5 (26.3)		
Timeliness	6 (4.2)	1 (5.3)	χ^2^(1) = 0.0491	0.825
Not	138 (95.8)	18 (94.7)		
No other dimension	144 (100)	19 (100)		

*n*, number of QI; QI, quality indicator; (%), % of all not-matching (or matching) values of the variable; all reported 163 QI; χ^2^, Pearson χ^2^ value with degrees of freedom in brackets ();

∗ Statistically significant (≤ 0.05).

### Prominent themes in National Anaesthesiologists Society reported quality indicators that do not match validated quality indicator sets

**Fig. 2:**
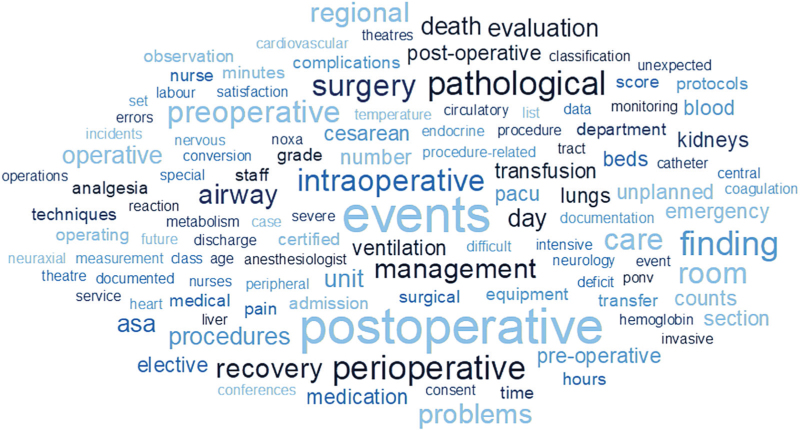
Word cloud of topics covered by National Anaesthesiologists Societies reported quality indicators not matching validated or well established quality indicator sets.

Aspects of care related to NAS-reported quality indicators that did not match validated quality indicator sets included relevant pathological findings related to specific organ systems; postoperative care, including intensive care, recovery and pain; patient blood management; staffing, including future shortages; and standards and protocols, among others (list of topics: SDC 4). Bearing in mind the methodological limitations of word clouds,^43^Fig. 2 provides a qualitative visual impression of the topics covered by ’mismatching’ quality indicators (excluding common words and generic terms, see above).

The word cloud was generated using MAXQDA. Applying a standard ‘stop word list’, the following generic terms were also excluded: ‘anaesthesia’, ‘anaesthesias’, ‘anaesthetic’, ‘anesthesia’, ‘fraction’, ‘general’, ‘indicator’, ‘patient’, ‘patients’, ‘reasons’, ‘related’, ‘relevant’, ‘standard’, ‘system’ and ‘undergoing’.

### Rating accuracy, inter-rater reliability and special observations

Initial agreement prior to consensus was mostly moderate for descriptive characteristics of quality indicators (Table 7). It was lower for quality attributes: Percentage agreement between raters ranged from 57.1 to 96.3%, and kappa coefficients showed low inter-rater reliability. For comparisons of NAS-reported quality indicators with validated quality indicator sets, percentage agreement ranged from 62 to 100%, but kappa coefficients showed only low or fair agreement.

**Table 7:** Inter-rater reliability

Measuring aspect	Two independent raters		Inter-rater reliability			
	Matches	Nonmatches	Percentage agreement	Cohen's kappa	95% CI	Strength of agreement
Descriptive characteristics						
Type of indicator	104	59	63.8	0.416	0.374 to 0.556	Moderate
Area of care	106	57	65.03	0.480	0.435 to 0.502	Moderate
Peri-operative phase	104	59	63.8	0.509	0.463 to 0.558	Moderate
Anaesthesia-specific or general	84	79	51.53	0.097	−0.03 to 0.224	Slight
Represented attributes of quality						
Appropriateness	130	33	79.75	0.477	0.325 to 0.629	Moderate
Availability/accessibility	117	46	71.78	−0.050	−0.166 to 0.066	Poor
Continuity	151	12	92.64	−0.021	−0.050 to 0.008	Poor
Effectiveness	125	38	76.69	0.219	0.049 to 0.390	Fair
Efficacy	136	27	83.44	0.280	0.079 to 0.480	Fair
Efficiency	142	21	87.12	0.021	−0.145 to 0.188	Slight
Prevention	130	33	79.75	0.237	0.051 to 0.423	Fair
Respect and caring	152	11	93.25	0.124	−0.145 to 0.393	Slight
Safety	93	70	57.06	0.140	−0.012 to 0.292	Slight
Timeliness	157	6	96.32	0.481	0.127 to 0.836	Moderate
Other dimension	155	8	95.09	0.000	0 to 1.000	Slight
Comparisons with validated/established QI sets						
StEP consensus clinical indicators	155	8	95.09	0.190	0.000 to 0.488	Slight
PSI patient safety indicators	143	20	87.73	0.309	0.142 to 0.485	Fair
Structure QI with high level of evidence	141	22	86.5	0.064	−0.021 to 0.218	Slight
Process QI with high level of evidence	148	15	90.8	0.038	0.002 to 0.093	Slight
StEP patient-centred outcomes	163	0	100	N/A	N/A	N/A
Principal requirements, ‘Helsinki Declaration’	101	62	61.96	0.180	0.113 to 0.214	Slight

CI, confidence interval; Helsinki Declaration, Helsinki Declaration on Patient Safety in Anaesthesiology^1^; N/A, not applicable; PSI, patient safety indicators^36^; QI, quality indicator. StEP, standardised endpoints in peri-operative medicine^28^.

In addition to calculating interrater agreement, notes on specific observations were taken during all consensus ratings and discussed in detail between the raters (detailed topics in Table 8).

**Table 8:** Issues of quality indicator concepts, definitions and practice (in-depth rater discussions)

Issues identified during ratings and discussed in-depth among raters	QI examples
General QI definition used by reporting organisation not clear	*‘No event (during anaesthetic care)’*
Unclear definition of what the QI measures (e.g. different aspects combined in one QI)	*‘General condition: nausea; vomiting; pruritus; insufficient analgesia; eye/corneal damage; unspecific headache; awareness during anaesthesia’*
Definition of follow-up period unclear	*‘Postoperative visit of the patient’*
Diverging definitions of mortality	*‘Death (postoperative/anaesthesia related events in the first 24h)’*
	*‘Death < 48h after anaesthesia’*
	*‘Intraoperative death’*
Time of relevant event related to QI not clearly defined for data collection	*‘Cardiac arrest’*
No information about underlying scientific evidence and validation of reported QI	*‘Are you performing parallel anaesthetics’*
Target audience of data unclear (internal quality improvement versus external reporting)	*‘Are you labelling syringes?’*
Practical details of data collection according to QI not clear (e.g. manual versus automated)	*‘Operative anaesthesia: Circulatory instability requiring catecholamines’*
Cycle of data collection unclear (prospective for every patient versus aggregated structure data)	*‘Preoperative – Preoperative haemoglobin optimisation’*
Quality control of data collection unclear (e.g. external audits)	*‘Neurological dysfunction < 3m after intervention, related to regional anaesthesia’*

QI, quality indicator.

## Discussion

### Summary

This study provides a comprehensive overview and analysis of quality indicators promoted by NAS in Europe. It shows that at the time, 12 of the 37 NAS made a quality indicator set available to their members, and in six of these countries, the collection of quality data was mandatory for anaesthetists. In 11 quality indicator sets shared with the study team, a total of 163 individual quality indicators were identified, most of which were descriptive, anaesthesia-specific, related to the intra-operative phase, and measuring mainly structural aspects of care. Commonly measured quality dimensions were safety, appropriateness and prevention. Only 11.7% of the quality indicators reported by the European NAS corresponded to previously published and validated quality indicators.

There are several strengths of the study. First, as it is based on the ‘grey literature’, that is a wide range of information published through different distribution channels such as NAS websites rather than in published articles, it extends knowledge in this field to unexplored and original data sources of quality indicators used in routine practice and includes non-English speaking countries. Second, the ESAIC network and close follow-up process helped to identify suitable respondents and achieve a 100% response rate. Third, the defined rating strategy and calculation of interrater agreement allowed us to assess the reliability of the overall analysis.

### Interpretation

Two previous systematic reviews identified 108 quality and safety indicators for anaesthesia^16^ and 1282 quality and safety indicators for clinical peri-operative structures and processes.^29^ Both reviewed quality indicators published in English, mostly in the scientific literature. Compared to 1% in one of these reviews,^16^ our study identified 41.7% of structure indicators (Table 2). This may be explained by the fact that, in contrast to previously published work on this topic,^16,29^ the quality indicators analysed in our study were based exclusively on ‘grey literature’,^16,29^ expert interviews and information from NAS websites. Thus, we were able to identify more quality indicators that relate to the regulatory aspects of anaesthesia care, are used on a daily basis and are less costly to collect and analyse. This is typically the case with structure indicators.^17^ However, there are drawbacks to this strategy. Structure indicators cannot measure all the different dimensions of quality of care^37^ and are unsuitable as proxies for outcome indicators^52^ because they do not relate to the care itself. In contrast, as in previous studies,^16^ we also found that the quality indicators reported by NAS were most often designed to measure the safety dimension and to be related to the intra-operative phase of care (Table 2). This indicates a need for quality measures for the postoperative phase of anaesthesia care.^53,54^

Furthermore, our study found a lack of clear definitions^55^ for several quality indicators promoted by the NAS (Table 8). For example, different definitions of the follow-up period for postoperative mortality may limit the comparability or benchmarking of outcomes.^20^ Second, combining several unrelated aspects into one quality indicator may lead to misleading results. Furthermore, we found that quality indicator definitions rarely included information about the process (e.g. audits) to ensure quality improvement when problems are identified, the data collection processes (for each patient or aggregated), the target audience (internal quality improvement versus external reporting^56^) and managing practical issues that may affect data collection by clinicians (i.e. legal concerns,^57,58^ time pressure, technical limitations or distractions^59^).

When benchmarking the NAS-reported quality indicators identified in this study, we found that only 11.7% of them corresponded to well established sets of validated measures. This confirms the findings of previous studies in this area, which reported a low or absent level of scientific evidence for most clinical quality indicators.^16,29,54,60^ The quality indicators that did match validated measure sets mostly measured the safety dimension of care (Table 6) and met several items of the Peri-operative Patient Safety Indicators ^36^ and the requirements of the Declaration of Helsinki on Patient Safety in Anaesthesiology^1^ (Table 4). They included common peri-operative care topics such as pre-operative antibiotic administration, surgical safety checklist, and morbidity and mortality registry or reporting (Table 5).

However, the quality indicators reported by NAS were rarely developed as patient-centred and value-based measures defined as ‘outcomes that matter to patients’.^61^ None of them matched the StEP patient-centred outcome measures,^35^ and only six (3.7%) related to ‘respect and caring’ (Table 3). This finding is not limited to quality measurement in anaesthesia but to the whole field of outcome measurement, where several other authors have found that patient-centred outcome measures (PCOM) have not been widely adopted in routine peri-operative care.^62,63^ This area is important because patient-centred outcomes, rather than physician perceptions, are increasingly used to determine medical success or failure^64^ and to define value^61^ and necessary expenditures in healthcare.^64,65^

We also found differences between countries in Europe in the provision of quality indicator by NAS to their members. This was more likely in countries in which reporting of quality data was mandatory for anaesthesiologists,^27^ which were located in the western or northern part of the continent and had a higher density of anaesthesia providers,^39,40^ and high WBGNI income.^41^ Quality indicator sets are also more often published on the internet by NAS in high-income European countries.^20,24,66–69^ In collaboration with NAS and other organisations, ESAIC initiatives such as the EQUIP project^27^ can contribute to the use and promotion of quality measurement and improvement at national and supranational levels.

This study provides a broad Europe-wide overview of quality measures and, as such, can contribute to improving patient outcomes and the quality of care^18,19^ and to reducing costs.^19^ To achieve such improvements, two aspects need to be closely considered. One is a continuing interest in the development and use of adequately risk-adjusted, validated peri-operative outcome measures, such as the StEP initiative and the WFSA indicator set to track timely access to safe surgical, anaesthetic and obstetric care.^20,70,71^ Another is to investigate the feasibility of using quality indicators in routine peri-operative practice and of linking^20^ data collected by various organisations, and to identify emerging issues of high importance to clinicians, patients and other healthcare stakeholders, such as issues related to staffing,^72,73^ handovers^74,75^ or failure to rescue.^76^ Finally, the gradual development of an ESAIC core set of quality indicators may be considered, as this could be a collaborative effort between national and supranational professional societies, research institutions, patient organisations and others.^16,77^ Delphi consensus methods^17,28,35,70^ and validation of the final core set of well defined, valid and feasible quality indicators should be considered.^78^

### Limitations

This study has several limitations. First, because we used the ESAIC organisation to identify NAS representatives, we may have contributed to a selection bias related to the varying levels of involvement of individual representatives in quality activities, potentially missing existing quality indicators. We attempted to minimise these shortcomings through multiple follow-ups or informal discussions where necessary. By design, our study may also have missed quality indicators used by organisations other than NAS. Second, the relatively long study period due to the chosen study design and the COVID pandemic may affect the timeliness of the results. However, as quality indicators relate more to the broader and regulatory dimension of care, timing is less likely to have influenced our results. Third, some transcription errors may have occurred. We attempted to minimise these through multiple double checks. Fourth, measurement bias may be associated with the ratings and the face-to-face consensus process. The comparatively low levels of interrater reliability (Table 7) prior to consensus indicate that many expert interactions were required to reach consensus. This may limit the internal validity and generalisability of the results; future studies may benefit from improving rater training and/or including intra-rater analysis.^49^ Fifth, the influence of ’training effects’ during ratings cannot be excluded. Finally, heterogeneous definitions and practical aspects of quality indicators posed a challenge to the analysis and may further limit generalisability of the results.

## Conclusion

The present study provides the first overview and characterisation of routinely used peri-operative quality indicators promoted by NAS in Europe, including non-English speaking countries. Such indicators were promoted by few NAS, differed mostly from previously published sets of validated quality measures, and did not evenly represent all dimensions of care quality. Further steps towards a valid and workable unique core set of routine peri-operative quality indicators should give priority to indicators that are well defined and validated, extend beyond the intra-operative phase, and measure outcomes and patient-centred aspects of care.

## Supplementary Material

Supplemental Digital Content
