# Supplementary material for: A survey and analysis of peri-operative quality indicators promoted by National Societies of Anaesthesiologists in Europe: The EQUIP project
Source: Eur J Anaesthesiol. 2024 Sep 12;41(11):800–12. doi: 10.1097/EJA.0000000000002054 (PMC11451932; doi:10.1097/EJA.0000000000002054)
Supplement: Supplemental Digital Content [file ejanet-41-800-s001.docx]

**1. TRANSLATION ISSUES**

On several occasions, the wording seemed unclear in the English translations. In cases where both reviewers had either first language proficiency or excellent second language proficiency of the respective original language, the wording was reviewed in the original language QI sets, compared to the translated sets, and adapted if it could be improved after consensus discussions (see methods section). The following adaptions were made:

After review of the QI set in the original language, the wording "*operative anaesthesia: Catecholamine-related circulatory instability*" was replaced by "*operative anaesthesia: Circulatory instability requiring catecholamines*” in one QI definition; the wording "*timely delivery (antibiotics)*" was replaced by "*timely administration (antibiotics)*” in another QI definition; the wording "*recognised training centre for anaesthetic medicine*” was changed to “*recognised training centre for anaesthesiology*” in the detailed descriptions of one QI; the wording “*operating theatres and theatre corridors*” was changed to “*operating theatres and theatre wings*” in another QI description; the wording of the description “*nursing staff (all; dipl…)*” was changed to “*nursing staff (all; certified…)*” in several other QI descriptions; the wording “*Post-operative follow-up*” was replaced by “*postoperative care*” and “*wake-up room*” by “PACU”; the wording “*emergency service*” was replaced by “*prehospital rescue service*”; after reviewing the context in the original language QI set, the wording “*respiratory*” was replaced by “*airway*” in several other QI descriptions; after reviewing the context in the original language QI set, the wording of "*number of requests for childbirth in analgesia not satisfied within 30 and 60 Minutes*" was replaced by "*number of requests for labour analgesia not satisfied within 30 and 60 Minutes*".
