# Supplementary material for: A survey and analysis of peri-operative quality indicators promoted by National Societies of Anaesthesiologists in Europe: The EQUIP project
Source: Eur J Anaesthesiol. 2024 Sep 12;41(11):800–12. doi: 10.1097/EJA.0000000000002054 (PMC11451932; doi:10.1097/EJA.0000000000002054)
Supplement: Supplemental Digital Content [file ejanet-41-800-s002.docx]

**2. WORKING DEFINITIONS OF VARIABLES FOR RATINGS**

***World Bank Gross National Income (per capita) Groups*^1^**

1. Low income ($1,035 or less)
2. Lower middle income ($1,036 - 4,045)
3. Upper middle income ($4,046 - 12,535)
4. High income ($12,536 or more)

***Physician Anesthesia Provider (PAP) Density - World Federation of Societies of Anaesthesiologists (WFSA)*^2,3^**

1. Physician anesthesia provider (PAP) density <10/100'000
2. PAP density 10-<20/100'000
3. PAP density 20-<30/100'000
4. PAP density >30/100'000

***Type of Indicator (Descriptive, Prescriptive, or Proscriptive)*^4^**

1. Descriptive (descriptive information on unusual situations of patient care that could reveal, if further investigated, potential defects in the quality of care provided - e.g., unplanned overnight admission of day surgery patients for anesthetic reasons)
2. Prescriptive (indicator represents recommendations or desired targets - e.g., prophylactic antibiotic selection for surgical patients according to current recommendations)
3. Proscriptive (measures of actions that “should not be performed” - e.g., medication error with the wrong medication being given)

***Area of Care (Process, Outcome, Structure – following Donabedian’s Model of Care Quality*^5^*)***

1. Process (indicator refers to implementation of program activities)
2. Outcome (indicator refers to patient-related end results of anesthesia (perioperative) care)
3. Structure (indicator refers to hospital staff, material, and overall organization)

***Perioperative Phase (Preoperative, Intraoperative, Postoperative,*^6^ *or “not Applicable”)***

1. Preoperative (from the decision to operate to entry into the theatre suite)
2. Intraoperative (from entry into the theatre suite to leaving the recovery area)
3. Postoperative (following transfer from the recovery area)
4. Not applicable

***Anesthesia-Specific or General*^4^**

1. Specific (indicator refers specifically to the practice of anesthesia)
2. General (indicator could also relate to surgical or postoperative ward care)

***Dimensions of Quality (10 Attributes, according to National Library of Healthcare indicators*^4^*). Each Attribute Represented an Individual Variable***

1. Appropriateness (the degree to which the care provided is relevant to the patient's clinical needs, given the current state of knowledge)
2. Indicator does NOT describe aspects of "Appropriateness"
3. Availability/accessibility (the degree to which appropriate care is available to meet the patient's needs)
4. Indicator does NOT describe aspects of "Availability/accessibility"
5. Continuity (the degree to which the care for the patient is coordinated among practitioners, organizations and over time)
6. Indicator does NOT describe aspects of "Continuity"
7. Effectiveness (the degree to which care is provided in a correct manner, given the current state of knowledge, to achieve the desired or projected outcome(s) for the patient)
8. Indicator does NOT describe aspects of "Effectiveness"
9. Efficacy (the degree to which the care of the patient has been shown to accomplish the desired or projected outcome(s))
10. Indicator does NOT describe aspects of "Efficacy"
11. Efficiency (the relationship between the outcomes (results of care) and the resources used to deliver patient care)
12. Indicator does NOT describe aspects of "Efficiency"
13. Prevention (the degree to which appropriate services are provided for promotion, preservation, and restoration of health and for early detection of disease)
14. Indicator does NOT describe aspects of "Prevention"
15. Respect and caring (the degree to which a patient, or designee, is involved in his or her own care decisions, and to which those providing services do so with sensitivity and respect for the patient’s needs, expectations and individual differences. This dimension represents patients’ perspectives)
16. Indicator does NOT describe aspects of "Respect and caring"
17. Safety (the degree to which adverse outcomes or injuries stemming from the processes of health care are reduced or avoided for the patient)
18. Indicator does NOT describe aspects of "Safety"
19. Timeliness (the degree to which the care is provided to the patient at the most beneficial or necessary time)
20. Indicator does NOT describe aspects of "Timeliness"
21. Other dimension
22. No obvious other dimension

***StEP Consensus Clinical Indicators (Set of 8 outcome indicators recommended as valid and reliable for use in perioperative clinical trials*^7^*)***

1. surgical site infection at 30 days
2. stroke within 30 days of surgery
3. death within 30 days of coronary artery bypass grafting
4. death within 30 days of surgery
5. admission to the intensive care unit within 14 days of surgery
6. readmission to hospital within 30 days of surgery
7. length of hospital stay (with or without in-hospital mortality)) - 2 definitions but both using day of surgery until discharge or death (see Haller et al.^7^)
8. NOT-COMPARABLE (to any of these indicators, in the raters view)

***PSI Clinical Indicators (a set of 11 perioperative patient safety indicators developed and clinically tested in hospitals*^8^*)***

1. Completion of the total STOP-bundle, % patients 7 separate stop moments: I. Preoperative risk management – screening the patient; II. Planning of the operation; III. Check of the current situation – checking whether all conditions for safe surgery are met; IV. Time-out before the operation; V. Sign-out after the operation; VI. Discharge from the recovery (predetermined discharge criteria; anaesthesiologist is responsible for performance/recording, discharge only when criteria); VII. Discharge from the hospital: (discharge only if medical condition permits this; destination capable of safe/adequate treatment. recorded: discharge decision, when, by whom; surgeon responsible)
2. Availability of a protocol on antibiotics
3. Timely administration of antibiotic prophylaxis (% patients)
4. AC-PROT (Availability of a protocol on anticoagulants)
5. Availability of a protocol on responsibilities regarding maintenance of medical equipment
6. Availability of a protocol on (performing) prospective risk analysis of medical equipment
7. Availability of OR-regulations
8. Presence of a surveillance system for postoperative wound infections
9. Postoperative wound infections (in-hospital) (%)
10. Presence of a morbidity and mortality registration
11. Postoperative mortality (in-hospital) (%)
12. NOT-COMPARABLE (to any of the 11 indicators, in the raters view)

***Structure QI with High level of Scientific Evidence*^6^ *(7 structure indicators (published in a recent systematic review*^6^) *that had a scientific level of 1, according to the levels of the Oxford Centre for Evidence-Based Medicine*^9^)**

1. There is a designated area suitable for private communication with patients. Patients are given adequate information upon which to base their decision about informed consent
2. Clinicians wishing to perform ultrasound guided regional anaesthesia should be experienced in the administration of regional nerve blocks and trained in ultrasound guidance techniques
3. Availability of surgical follow up within 30 days following hospital discharge
4. Hospital annual case volume
5. Protocols exist for the perioperative management of: venous thromboembolism prophylaxis, avoidance of hypothermia, management of diabetes mellitus, handover, anaesthetic emergencies, morbidly obese patients, handling of complaints, elderly patients, remote site anaesthesia, end of life care, and critical care referral
6. Surgical monthly/annual case volume by surgical specialty
7. Bed size of hospital: how many adult inpatient/overnight/23 h stay available within the hospital
8. NOT-COMPARABLE (to any of the 7 indicators, in the raters view)

***Process Indicators with High Levels of Scientific Evidence*^6,8^ *(35 process indicators*^6^ *published in a recent systematic review that had a scientific level of 1*^9^*)***

1. Percentage of patients who have received an anaesthetic assessment before the day of surgery
2. Each patient should have his or her expected risk of death estimated and documented prior to intervention and due adjustments made in urgency of care and seniority of staff involved
3. For alcohol abusers 1 month abstinence before surgery. For daily smokers, 1 month abstinence before surgery. Offer smoking cessation advice
4. Adequate preoperative fasting: clear fluids up to 2 h prior to surgery, solids up to 6 h prior to surgery
5. Patient nutritional status assessed within 48 h of admission to hospital by a dietician
6. No routine administration of preoperative anaesthetic medication or sedation
7. Mechanical bowel prep not used routinely for colonic surgery
8. What proportion of patients had preoperative prophylactic venothromboembolism therapy?
9. Preoperative oral carbohydrate treatment used routinely for all non-diabetic patients
10. Prophylactic antibiotics are administered within 60 min before start of surgery
11. Adults having surgery under general or regional anaesthesia have normothermia (temperature >36.0°C) maintained before, during, and after surgery
12. Proportion of patients who have had appropriate prophylactic antibiotic selection for surgical patients
13. An appropriately trained and experienced anaesthetist is present throughout the conduct of all general and regional anaesthesia for operative procedures
14. If hair removal is required, it should not be performed with a razor but with clippers
15. A multimodal approach for postoperative nausea and vomiting prophylaxis should be adopted in all patients with ≥2 risk factors
16. Optimized perioperative fluid management: targeting cardiac output, avoiding over-hydration, and judicious use of vasopressors. Targeted fluid therapy using the Doppler is recommended
17. Patients for whom a central venous catheter was inserted with all elements of sterile barrier technique followed Documentation of daily examination of line site for signs of infection and continued need for central line
18. Epidural analgesia used intraoperatively
19. Surgical field preparation with chlorhexidine-alcohol
20. Adequate perioperative management of patient's current medications
21. No systemic morphine used intraoperatively
22. Proportion of surgical patients who had an order for venous thromboembolism prophylaxis to be given within 24 h before incision/after surgery end
23. Proportion of patients whose prophylactic antibiotics were discontinued within 24 h after surgery end time
24. Patients should be encouraged to sit out of bed and begin mobilising the day after surgery, within 24 h or as determined by the surgeon
25. Discharge needs assessment, venous thromboembolism prophylaxis, rehab, and follow up are organized postoperatively for patients
26. Urinary catheter removed on Postoperative Day 1 or Postoperative Day 2 with day of surgery being Day 0, or reason for continuing use documented
27. Postoperative treatment of diabetes mellitus (or documentation of attempt) to keep BM <10 mmol litre−1 on day of surgery and the first 2 postoperative days
28. Postoperative nasogastric tubes should not be used routinely
29. Enteral route for postoperative fluid used as soon as possible, i.v. fluids discontinued as soon as is practicable
30. Postoperative delirium screening for all patients
31. Postoperative normothermia maintained at: 36–38°C
32. Stimulation of bowel movements using an even fluid balance, laxatives and chewing gum
33. Chronic beta blocker use is continued in perioperative period (24 h before incision to first 2 postoperative days)
34. Percentage of surgery patients who received appropriate venous thromboembolism prophylaxis within 24 h prior to surgery to 24 h after surgery
35. Enhanced recovery used perioperatively
36. NOT-COMPARABLE (to any of the 35 indicators, in the raters view)

***StEP Patient Centered Outcomes (for use in clinical trials)*^10^**

1. EuroQol 5 Dimension (health- related quality-of-life measure)
2. WHO Disability Assessment Schedule version 2.0, 12-question version (functional-status measure)
3. Days alive and out of hospital at 30 days after surgery (life-impact measure)
4. NOT-COMPARABLE (to any of the 3 patient-centered outcome measures, in the raters view)

***Principal Requirements of the Helsinki Declaration on Patient Safety in Anaesthesiology*^11^ *(patient safety requirements resembling structure QIs that are generally well accepted in anaesthesia*^11^*)***

1. Comply with minimum standards of monitoring recommended by the EBA (OR / recovery). 1.1 Anaesthesia: SpO2, NIBP, ECG, O2/CO2/vapour analyzers, Airway pressure, nerve stim, Temp, Stethoscope
2. Comply with minimum standards of monitoring recommended by the EBA (OR / recovery). 1.2. Recovery: SpO2, NIBP, ECG, (CO2), nerve stim., Temp
3. Should have protocols, and the necessary facilities for managing the following: 2.1. Preoperative assessment and preparation
4. Should have protocols, and the necessary facilities for managing the following: 2.2. Checking Equipment and drugs
5. Should have protocols, and the necessary facilities for managing the following: 2.3. Syringe labelling
6. Should have protocols, and the necessary facilities for managing the following: 2.4. Difficult/failed intubation
7. Should have protocols1,4, and the necessary facilities for managing the following: 2.5. Malignant hyperpyrexia
8. Should have protocols, and the necessary facilities for managing the following: 2.6. Anaphylaxis
9. Should have protocols, and the necessary facilities for managing the following: 2.7. Local anaesthetic toxicity
10. Should have protocols1,4, and the necessary facilities for managing the following 2.8. Massive haemorrhage
11. Should have protocols, and the necessary facilities for managing the following: 2.9. Infection control
12. Should have protocols, and the necessary facilities for managing the following: 2.10. Postoperative care including pain relief
13. Institutions providing sedation: comply with anaesthrdiology recognised sedation standards for safe practice
14. Support WHO SSSL initiative and Checklist
15. Annual safety report: measures taken and results obtained in improving patient safety locally
16. Collect the required data to be able to produce an annual report on patient morbidity and mortality.
17. Contribute to recognised audits of safe practice and critical incident reporting systems. Resources must be provided to achieve this.
18. Covering MULTIPLE HD requirements, in the raters view
19. NOT-Covering (any of the HD requirements, in the raters view)

**REFERENCES**

1 The World Bank. *World Bank List of Economies (June 2020)*. Washington, DC, USA: The World Bank, 2020.

2 Kempthorne P, Morriss WW, Mellin-Olsen J, Gore-Booth J. The WFSA global anesthesia workforce survey. *Anesth Analg* 2017; **125**:981–990.

3 World Federation of Societies of Anesthesiologists WFSA. *World Anaesthesiology Workforce Map*. London, UK: WFSA, 2023.

4 Haller G, Stoelwinder J, Myles PS, McNeil J. Quality and safety indicators in anesthesia: a systematic review. *Anesthesiology* 2009; **110**:1158–1175.

5 Donabedian A. The quality of care. How can it be assessed? *JAMA* 1988; **260**:1743–1748.

6 Chazapis M, Gilhooly D, Smith AF et al. Perioperative structure and process quality and safety indicators: a systematic review. *Br J Anaesth* 2018; **120**:51–66.

7 Haller G, Bampoe S, Cook T et al. Systematic review and consensus definitions for the Standardised Endpoints in Perioperative Medicine initiative: clinical indicators. *Br J Anaesth* 2019; **123**:228–237.

8 Emond YE, Stienen JJ, Wollersheim HC et al. Development and measurement of perioperative patient safety indicators. *Br J Anaesth* 2015; **114**:963–972.

9 Phillips B, Ball C, Sackett D et al. Oxford centre for evidence-based medicine: levels of evidence (march 2009). 2009. https://www.cebm.ox.ac.uk/resources/levels-of-evidence/oxford-centre-for-evidence-based-medicine-levels-of-evidence-march-2009 [accessed 13 June 2022].

10 Moonesinghe SR, Jackson AIR, Boney O et al. Systematic review and consensus definitions for the Standardised Endpoints in Perioperative Medicine initiative: patient-centred outcomes. *Br J Anaesth* 2019; **123**:664–670.

11 Mellin-Olsen J, Staender S, Whitaker DK, Smith AF. The helsinki declaration on patient safety in anaesthesiology. *Eur J Anaesthesiol* 2010; **27**:592–597.
