# Supplementary material for: A survey and analysis of peri-operative quality indicators promoted by National Societies of Anaesthesiologists in Europe: The EQUIP project
Source: Eur J Anaesthesiol. 2024 Sep 12;41(11):800–12. doi: 10.1097/EJA.0000000000002054 (PMC11451932; doi:10.1097/EJA.0000000000002054)
Supplement: Supplemental Digital Content [file ejanet-41-800-s003.docx]

**3. NAMES OR TITLES OF 163 QIS REPORTED BY NAS**

The following list presents the names or titles of QI as reported by NAS. In accordance with the anonymity requirements of the study design, QIs are presented in alphabetical order (irrespective of reporting by NAS). In addition, specific terms that could have allowed identification of individual NAS were replaced by non-specific terms in square brackets [].

Abbreviations: ASA, American Society of Anesthesiologists; CIRS, critical incident reporting system; ESA (now ESAIC), European Society of Anaesthesiology (and Intensive Care); ICU, intensive care unit; NAS, national anaesthesiologists’ society; NRS, numerical rating scale (pain level); OPD, outpatient department; OT, operating theatre; PACU, post-anaesthesia care unit; PONV, perioperative nausea and vomiting; M&M, morbidity and mortality; RA, regional anaesthesia; VAS, visual analogue scale (pain level).

% of patients undergoing Day Surgery hospitalized on a regular admission basis for anaesthetic reasons

% patients deleted from the surgical list on the day of surgery for anaesthetic reasons

% patients with need for postoperative anaesthetic assistance more than 6 hours after Day Surgery

Airway management: Fraction of patients with impossible mask ventilation, indicator standard <0.16%

Airway management: Fraction of patients with unanticipated difficult airway management, indicator standard <6%

Airway, lungs (events)

Airway, lungs (events)

Anaesthesia for all kinds of procedures

Anaesthesia related problems - Airway related

Anaesthesia related problems - Death during anaesthesia

Anaesthesia related problems - Other anaesthesia related problems

Anaesthesia related problems - Regional anaesthesia related problems

Anaesthetic recovery room: Aldrete score >8

Anaesthetic recovery room: Neurological abnormalities central/ peripheral

Anaesthetic recovery room: Shivering

Anaesthetic recovery room: Vomiting (PONV)

Anaphylactic shock

Antibiotics given within the hour (preferably 30 min) before skin incision

Are equipment stationary or mobile?

Are You analyzing critical accidents?

Are You labelling syringes?

Are You performing parallel anaesthesias

Aspiration

Blood system and coagulation: Is there a relevant pathological finding with regard to the blood system/coagulation?

Cardiac arrest

Cardiovascular (events)

Cardiovascular (events)

Chronic pain service

Circulation: Is there a relevant pathological finding for the circulatory system?

Complications

core data set anaesthesia 3.x (from standard anaesthesia protocols)

Counts by age

Counts by ASA score

Counts emergency/elective

Counts of procedures (e.g., epidurals, central venous and arterial cannulations, etc)

day case surgery beds

Death

Death

Death < 48h after anesthesia

Demography, ASA class: Height; Weight; ASA class

[NAS] recommendations medical staffing

Different kind of anaesthesia numbers (anaesthesia types/subtypes)

Do You have critical accident reporting?

Do You have guidelines ESA, [NAS] available?

Do You have induction room, PACU?

Do You have surgical check-list?

Do You have Your own protocols?

Doctor/nurse/patient

Equipment

Events during anaesthetic care: procedure-related, anaesthesia

Events during anaesthetic care: procedure-related, operation

Existence and use of a list for the daily control of ventilation equipment (respirator)

For how many patient You may perform ventilation?

Further tasks of your department

Future deficit of nurses

Future deficit: (how many) are going to retire; other reasons

General condition (postoperative)

handover- and/or discharge protocols as proposed in the Helsinki Declaration

Heart: Is there a relevant pathological finding for the heart?

Hip fracture surgery performed within 48 hours from admission

Incidence of post-anaesthesia headache--- neuraxial analgesia with compatible characteristics

Intraoperative - Anesthesia equipments checklist

Intraoperative - Antibiotic prophylaxis

Intraoperative - Difficult airways units

Intraoperative - Incidents Notification system

Intraoperative - Medication according [NAS]

Intraoperative - One anesthesiologist/one nurse anesthesiologist / one anesthesia ratio

Intraoperative - PONV prophylaxis

Intraoperative - Surgical safety checklist

Intraoperative - Temperature registry

Intraoperative death

Kidneys (events)

Kidneys (events)

Kidneys: Is there a relevant pathological finding for the kidneys?

Lungs / respiratory tract: Is there a relevant pathological finding for the lungs?

management od medical errors (CIRS reporting and/or M&M conferences and/or documented case conferences

Medication (events, errors); transfusion reaction

Medication (events), transfusion reaction

Medication / Device error

Metabolism and endocrine system: Is there a relevant pathological finding with regard to the metabolism/endocrine system?

Monitoring, special techniques: Vessel catheter (only if inserted recently); Invasive monitoring; Special techniques

N of beds

N of surgical beds

Nervous system (events)

Nervous system (events)

Neurologic dysfunction < 3m after intervention, related to regional anesthesia

Neurology: Is there a relevant pathological finding with regard to the neurology?

No event

Noxa: Are the relevant noxa present?

Number of complaints per 1000 anaesthetic procedures

Number of labour epidural analgesia/Number of vaginal births

Number of requests for labour analgesia not satisfied within 30 and 60 Minutes

Nurses anaesthetists: working; certified; non certified; nurse assistants

Obstetric beds

Operating Sessions ended after the scheduled time (>60 minutes)

Operating theatres and theatre wings

Operating theatres: Tables; manipulation rooms; CT, MRI; Endoscopy; PACU; Anaesthesias outside op. Theatre

Operative anaesthesia: Anaesthetic procedures (AN, neuroaxial procedures, peripheral RA)

Operative anaesthesia: Change in the anaesthetic procedure

Operative anaesthesia: Circulatory instability requiring catecholamines

Operative anaesthesia: Patient Blood Management (new)

Operative anaesthesia: pre-operative timely administration (30 min pre-op) (??antibiotics??)

Operative anaesthesia: Resuscitation

Operative anaesthesia: unplanned transfer to the ICU

other (beds?)

pain measurement on the ward, at least 3 times a day NRS taken and documented

patient blood management

Percentage of caesarean sections conducted under neuraxial anaesthesia

Perioperative procedural time

Perioperative: Fraction of patients undergoing elective cesarean section during general anesthesia, indicator standard <5%

Perioperative: Fraction of patients undergoing emergency cesarean section (grade 1–3) during general anesthesia, indicator standard <15%

Perioperative: Fraction of patients undergoing emergency cesarean section with regional anesthesia requiring a conversion to general anesthesia (grade 1–3), indicator standard <1%

Perioperative: Fraction of patients with anesthesia related complications, indicator standard <3.5%

Perioperative: Fraction of patients with insufficient regional anesthesia, indicator standard <3%

Perioperative: Fraction of patients with non-emergency bleeding where hemoglobin level is measured prior to erythrocyte transfusion, indicator standard =100%

Perioperative: Fraction of patients with regional anesthesia undergoing elective cesarean section requiring a conversion to general anesthesia (emergency grade 4), indicator standard <1%

Post-medication visits: No event, progress without complications

Post-operative care

Post-operative care (up to max. 72h): RA- catheter support (1x/24h)

Postoperative - Morbidity and mortality registry

Postoperative - Patient satisfaction

Postoperative - Post anesthetic care units (PACU)

Postoperative analgesia: Methods; Drugs; Do You have multimodal analgesia protocols? Do You use NRS, VAS??

postoperative visit of the patient

Postoperative: Fraction of patients arriving at the postoperative observation unit with hypothermia (<36°C) after a duration of anesthesia >2 hours, indicator standard <5%

Postoperative: Fraction of patients with nausea requiring treatment in the postoperative observation unit, indicator standard <2%

Postoperative: Fraction of patients with severe postoperative pain (numeric rating scale >7) in the postoperative observation unit, indicator standard <5%

Pre-operative patient evaluation for anaesthesia: ASA Classification

Pre-operative patient evaluation for anaesthesia: Categorisation [according to national medical services catalogue] into minor or severe operations according to the main guideline for pre-operative patient evaluation

Pre-operative patient evaluation for anaesthesia: Determining the metabolic equivalents (METS)

Prehospital rescue service

Preoperative - Anesthesia evaluation in the day before the procedure

Preoperative - Anesthesia medical appointment

Preoperative - Informed consent approval and signature

Preoperative - Preoperative hemoglobin optimization

Preoperative - Thromboembolic risks evaluation.

Preoperative: Fraction of patients without information of preoperative risk factors, indicator standard <5%

Presence of a surveillance system for anaesthetic incidents

Presence of anaesthetic documentation containing the minimum set of data identified in the requirements for accreditation

Presence of anaesthetic examination documentation for elective surgery

Presence of the Anaesthesia Consent Documentation for elective surgery

Procedure related (events: transfusion)

reporting about perioperative mortality and morbidity on a yearly basis as well as measures for improvement

Safety protocols as proposed in the Helsinki Declaration

Setting, Type of anaesthesia department

Staff: Age groups; Certified; Residents

Staff: Doctors N

Stomach and liver: Is there a relevant pathological finding for the gastrointestinal tract/liver?

Surgical operations on patients stratified according to the American Society of Anaesthesiologists’ (ASA) classification

Techniques of anaesthesia

temperature management

The proportion of patients who were discharged from a recovery room when they reported a VAS score of 3 or less

The rate of operated patients under general or regional anesthesia that their surgery lasts for more than an hour and the temperature on arrival to recovery room was 35.5°C.

timely administration (30-60 min before incision) of antibiotics in those cases needing antibiotics

To measure from the time of termination of anesthesia but not to disqualify cases in which the first measurement took place more than 15 minutes after anesthesia ended.

Total actual staff of entire department

Unexpected difficult intubation

Unexpected resp failure necessitating (non)invasive ventilation prior to discharge from PACU

Unplanned (post-operative) transfer: transfer to the intensive care unit (24h post-op), unplanned hospitalisation of an OPD patient

Unplanned post-operative transfer (to the intensive care unit; directly from OT)

Unplanned postoperative admission <24h to ICU, related to anesthesia

User satisfaction survey system

WHO-Safe-Surgery Checklist
