# Supplementary material for: A survey and analysis of peri-operative quality indicators promoted by National Societies of Anaesthesiologists in Europe: The EQUIP project
Source: Eur J Anaesthesiol. 2024 Sep 12;41(11):800–12. doi: 10.1097/EJA.0000000000002054 (PMC11451932; doi:10.1097/EJA.0000000000002054)
Supplement: Supplemental Digital Content [file ejanet-41-800-s004.docx]

**4. WORD FREQUENCY ANALYSIS OF “NON-MATCHING” QI TITLES/NAMES**

In order to assess topics represented by NAS indicators that were not identified in existing published sets of indicators, lists of single words and 2-word combinations (minimum frequency: 2) were generated using MAXQDA® (see main text for methodological details).

Abbreviations: ASA: American society of anesthesiologists; PACU: post anesthesia care unit; PONV: postoperative nausea and vomiting, QI, quality indicator

QI titles/names Frequency

Single words

Anaesthesia 25

Patients 19

Anesthesia 18

Anaesthetic 14

Standard 14

Events 13

Fraction 13

Indicator 13

Postoperative 13

Patient 11

Related 11

System 10

Relevant 9

Finding 8

Pathological 8

Perioperative 8

Care 7

Intraoperative 7

Preoperative 7

Room 7

Surgery 7

Airway 6

General 6

Management 6

Problems 6

Recovery 6

Regional 6

ASA 5

Day 5

Death 5

Evaluation 5

Operative 5

Procedures 5

Undergoing 5

Unit 5

Beds 4

Blood 4

Cesarean 4

Counts 4

Elective 4

Emergency 4

Kidneys 4

Lungs 4

Medication 4

Number 4

PACU 4

Post-operative 4

Pre-operative 4

Section 4

Transfusion 4

Unplanned 4

Ventilation 4

Admission 3

Analgesia 3

Certified 3

Complications 3

Department 3

Equipment 3

Grade 3

Hours 3

Medical 3

Minutes 3

Nurse 3

Observation 3

Operating 3

Pain 3

Protocols 3

Reasons 3

Score 3

Staff 3

Surgical 3

Techniques 3

Time 3

Transfer 3

Age 2

Anaesthesias 2

Anesthesiologist 2

Cardiovascular 2

Case 2

Catheter 2

Central 2

Circulatory 2

Class 2

Classification 2

Coagulation 2

Conferences 2

Consent 2

Conversion 2

Data 2

Deficit 2

Difficult 2

Discharge 2

Documentation 2

Documented 2

Endocrine 2

Errors 2

Event 2

Future 2

Heart 2

Hemoglobin 2

Incidents 2

Intensive 2

Invasive 2

Labour 2

List 2

Liver 2

Measurement 2

Metabolism 2

Monitoring 2

Nervous 2

Neuraxial 2

Neurology 2

Noxa 2

Nurses 2

Operations 2

Peripheral 2

PONV 2

Procedure 2

Procedure-related 2

Reaction 2

Satisfaction 2

Service 2

Set 2

Severe 2

Special 2

Temperature 2

Theatre 2

Theatres 2

Tract 2

Unexpected 2

Word combinations

Indicator standard 13

Pathological finding 8

Relevant pathological 8

Perioperative fraction 7

Anaesthesia related 6

Recovery room 6

Related problems 6

Operative anaesthesia 5

Regional anesthesia 5

Anaesthetic recovery 4

Cesarean section 4

General anesthesia 4

Patient evaluation 4

Patients undergoing 4

Pre-operative patient 4

Airway management 3

Anesthesia indicator 3

Observation unit 3

Postoperative fraction 3

Postoperative observation 3

Airway lungs 2

Anaesthetic care 2

Anaesthetic procedures 2

Anaesthetic reasons 2

ASA class 2

ASA classification 2

Blood management 2

Blood system 2

Cardiovascular events 2

Care procedure-related 2

Care unit 2

Day surgery 2

Elective cesarean 2

Emergency cesarean 2

Endocrine system 2

Events airway 2

Events kidneys 2

Events nervous 2

Events transfusion 2

Events unplanned 2

Future deficit 2

Intensive care 2

Kidneys events 2

Lungs events 2

Management fraction 2

Medication events 2

Monitoring special 2

Nervous system 2

Operating theatres 2

Patient blood 2

Post-operative care 2

Post-operative transfer 2

Problems anaesthesia 2

Reaction cardiovascular 2

Reasons patients 2

Special techniques 2

System events 2

Transfusion reaction 2

Undergoing elective 2

Undergoing emergency 2

Unit indicator 2

Unplanned post-operative 2
